# Chemotherapy for high-grade gliomas

**DOI:** 10.1054/bjoc.1999.1075

**Published:** 2000-03-21

**Authors:** E Galanis, J Buckner

**Affiliations:** Division of Medical Oncology, Mayo Clinic and Foundation, 200 First Street SW, Rochester, MN, 55905, USA

**Keywords:** chemotherapy, high-grade gliomas, glioblastoma multiforme, anaplastic astrocytoma, oligodendroglioma, oligoastrocytoma


*British Journal of Cancer* (2000) **82**(8), 1371–1380
© 2000 Cancer Research Campaign
DOI: 10.1054/ bjoc.1999.1075, available online at http://www.idealibrary.com on IDEAL®

Review

### Chemotherapy for high-grade gliomas

**E Galanis and J Buckner**

Division of Medical Oncology, Mayo Clinic and Foundation, 200 First Street SW, Rochester, MN 55905, USA

**Keywords:** chemotherapy; high-grade gliomas; glioblastoma multiforme; anaplastic astrocytoma; oligodendroglioma; oligoastrocytoma

High-grade gliomas are malignant neoplasms with high propensity for local recurrence. Despite combined modality treatment including surgical resection and radiation, a minority of patients live beyond 2 years and for tumours such as glioblastoma multiforme, the median survival is less than 1 year (Lesser and Grossman, 1994). Since the introduction of nitrosoureas in the early 1970s, a large number of clinical trials have examined the role of chemotherapy both after surgery and in the setting of recurrent disease. Frequently disappointing results, as discussed in this review, led to questioning of the role of chemotherapy in the management of patients with high-grade gliomas.

Improvement of chemotherapeutic options in neuro-oncology is hampered by both the efficacy of the available agents as well as methodological problems. The most obvious obstacle to effective chemotherapy for high-grade gliomas remains the intrinsic chemoresistance of these tumours to available agents (Petersdorf and Berger, 1996). The role of the blood–brain and blood–tumour barriers in preventing adequate drug concentration in the tumour remains uncertain (Mak et al, 1995). While lipophilic small molecules with low-binding fraction to the serum proteins seem to be better suited for this purpose, the role of blood–brain barrier was most likely overemphasized in the past. This is indirectly suggested by the increased contrast enhancement of high-grade gliomas, indicating a defective blood–brain barrier. In addition, high response rates achieved with agents that do not easily cross the blood–brain barrier, such as cisplatin in patients with chemosensitive central nervous system (CNS) tumours, i.e. embryonal tumours of the CNS (Galanis et al, 1997) and germ cell tumours (Sebag-Montefiore et al, 1992) underscore the importance of chemoresponsiveness rather than blood–brain barrier as being a major determinant of brain tumour response to chemotherapy. In addition, the pharmacokinetics of agents employed for the treatment of high-grade gliomas can be affected in patients who are on anticonvulsants as a result of the induction of hepatic oxidation and activation pathways (such as cytochrome p450). These drug interactions may lead to suboptimal drug concentrations as has been shown for paclitaxel (Chang et al, 1998), 9-aminocamptothecin (Grossman et al, 1998) and dacarbazine (Safgren et al, 1998).

Another fundamental question that has not yet been fully addressed is whether or not conventional oncologic criteria of response are useful and true measures of chemotherapy efficacy in gliomas. The poor correlation between responses measured in phase II studies and survival in adjuvant studies suggest that this methodology may be problematic. A definition of response similar to that for tumours outside the CNS provides a common language; however, it is not clear if tumour response translates into improvement in duration or quality of patient survival. Factors which affect interpretation of response in the brain include prior surgery, radiation therapy, corticosteroids and variability in scan techniques. It is possible that the criteria currently in use may not measure true tumour response to the same extent in the brain as in tumours outside the CNS. Alternatively, high-grade gliomas are not particularly chemosensitive tumours and it may be that we accept too low a threshold of response as evidence of efficacy (Brada and Sharpe, 1996). With these points in mind, we will review the available data on chemotherapy for high-grade gliomas in the adjuvant/neoadjuvant setting and for recurrent disease. The focus of this review is to look critically at the available evidence in order to determine the optimal use of chemotherapy for high-grade glioma patients. In addition, we will expand on newer investigational directions that may lead to advances in future treatments.

### GLIOBLASTOMA MULTIFORME/ANAPLASTIC ASTROCYTOMA: ADJUVANT CHEMOTHERAPY

Unlike adjuvant treatment in other oncological fields, adjuvant chemotherapy in high-grade gliomas does not imply the absence of residual disease at the time of treatment. Furthermore, adjuvant treatment does not attempt to prevent distant metastases but rather aims toward better local control. Introduction of the nitrosoureas in the 1970s remains the earliest and most significant chemotherapy contribution to the treatment of malignant glioma to date. Two groups of investigators simultaneously reported phase II data indicating that BCNU is active in patients with recurrent tumours,

*Received 13 January 1999*
*Revised 15 July 1999*
*Accepted 15 November 1999*

*Correspondence to:* E Galanis

**Table 1** Phase III trials of adjuvant therapy of malignant gliomas

| Treatment | *n* | Results | Reference |
|---|---|---|---|
| BTSG Trial 6901<br>BCNU<br>RT<br>BCNU + RT<br>Supportive care | 222 | Improved survival for patients receiving RT and BCNU + RT vs BCNU or best supportive care | Walker et al, 1978 |
| BTSG Trial 7702<br>RT + BCNU<br>RT + misonidazole + BCNU<br>RT + streptozotocin<br>Hyperfractionated RT + BCNU | 557 | No significant difference among the arms | Deutsch et al, 1989 |
| BTSG Trial 8001<br>RT + BCNU<br>RT + BCNU/procarbazine<br>RT + BCNU + hydroxyurea/procarbazine + VM-26 | 510 | No significant difference among the arms | Shapiro et al, 1989 |
| RTOG/ECOG Trial 7401<br>RT<br>RT + RT boost<br>RT + BCNU<br>RT + MeCCNU + DTIC | 554 | No significant difference among the arms. Overall improved survival in patients 40–60 years with chemotherapy + RT | Chang et al, 1983; Nelson et al, 1988 |
| NCCTG<br>RT + PCNU<br>RT + BCNU | 346 | No significant difference among the arms. BCNU – more haematologic toxicity. PCNU – more GI toxicity | Dinapoli et al, 1993 |
| RTOG 7918<br>RT + BCNU<br>RT + misonidazole + BCNU | 293 | No significant difference between the arms. Misonidazole produced peripheral neuropathy | Nelson et al, 1986 |
| SW0G Trial 7703<br>RT + BCNU<br>RT + Procarbazine<br>RT + Dacarbazine (DTIC) | 278 | BCNU + DTIC arms had better response rates compared to procarbazine arms (39 and 38% vs 13%). No statistically significant difference in survival | Shapiro et al, 1992 |
| SWOG Trial 7404<br>RT + CCNU<br>RT + CCNU + Procarbazine | 115 | No significant difference between the arms | Eyre et al, 1983 |
| NCCTG<br>RT + BCNU<br>RT + dibromodulcitol (DBD, halogenated hexitol functioning as alkylator) | 238 | Somewhat higher but no statistically significant different failure rates in the DBD arm | Elliott et al, 1997 |

albeit in the era prior to computerized tomography (CT) or magnetic resonance imaging (MRI) (Walker and Hurwitz, 1970; Wilson et al, 1970). Subsequently, the Brain Tumor Study Group (BTSG) in 1978 reported the results of a clinical trial (BTSG 6901) comparing the best conventional supportive care with carmustine-[1,3-bis (2-chloroethyl)-1-nitrosourea (BCNU)] and radiotherapy (RT) either alone or in combination. The median survival of the patients in the support-only group was 14 weeks as compared to 19 weeks in the BCNU-alone group, 36 weeks in the RT-alone group and 35 weeks in the RT/BCNU group. Survival curves for the patients receiving radiotherapy alone and for those receiving carmustine plus radiation therapy were not significantly different. Although survival distribution curves were practically identical for the first 12 months from initiation of treatment, there was a greater survival rate at 18 months among the patients receiving the combination of carmustine plus radiotherapy with 10% still alive at that time, as compared to only 4% of patients in the radiation-alone group (Walker et al, 1978). The BTSG trial 7201 confirmed the survival advantage that the combination of RT and nitrosourea-based chemotherapy offered at 18 months. In that study, methyl CCNU (semustine) alone proved to be significantly worse as compared to RT, RT + BCNU, and RT + meCCNU (Walker et al, 1980). However, the difference between nitrosourea/RT arms and radiation alone was not statistically significant, possibly secondary to small sample size, and no difference in median survival was observed. The BTSG trial 7501 randomized patients to receive in addition to 6000 rads of radiotherapy one of four treatment regimens: carmustine (BCNU), high-dose methylprednisolone (400 mg/m$^2$ per day for 7 days every 3 weeks), procarbazine, or BCNU + high-dose methylprednisolone. With the exception of RT + high-dose methylprednisolone, all other arms were equivalent in terms of median survival and percentage of patients alive at 18 months (Germain and Margulies, 1993). Thus, this study proved that high-dose steroids do not have an antitumour effect and that using steroids in amounts considerably higher than those required for elimination of cerebral oedema and symptom control does not increase survival.

Levin et al (1990) performed a phase III comparison of BCNU and the combination of procarbazine, CCNU and vincristine (PCV) administered after radiotherapy with hydroxyurea for

 *© 2000 Cancer Research Campaign*

malignant gliomas. A total of 133 patients were randomized; 73 with anaplastic gliomas and 60 with glioblastoma multiforme (29 at the BCNU arm, 31 at the PCV arm). The PCV combination produced longer overall survival and time to tumour progression than BCNU for both histologic groups, although the difference was statistically significant only for the anaplastic gliomas. Median survival was 82.1 weeks in the BCNU arm versus 157.1 weeks in the PCV arm. Prognostic covariates such as age, performance status and extent of surgical resection were balanced between the two histologic strata and two treatment groups. One of the pitfalls of this trial, however, is the inclusion of oligodendroglial-containing tumours in addition to anaplastic astrocytomas. Subsequently, in patients with anaplastic astrocytomas, the RTOG 9404 trial compared RT + PCV versus RT + PCV + the radiosensitizer BUdR. So far no difference in survival between the two arms has been detected (Prados et al, 1997).

Additional phase III adjuvant chemotherapy studies that have failed to show survival benefit of other chemotherapy regimens compared with BCNU + RT are summarized in Table 1. Of note in all of these trials, anaplastic astrocytomas were grouped together with glioblastoma multiforme.

The Medical Research Council recently reported in an abstract form on a prospective, randomized trial of adjuvant radiation therapy versus radiation therapy plus PCV in high-grade glioma patients < 70 years of age (MRC-BR05). With a median follow-up of 1 year and 674 patients enrolled, there was no significant difference in median survival between the radiation therapy and the combined treatment arm (Bleehen et al, 1998).

Of note, in all the prospective randomized high-grade glioma adjuvant therapy trials, where RT alone was one of the treatment arms, there was either no difference between the RT alone and the RT plus nitrosourea arms (i.e. BTSG trial 7201 (Walker et al, 1980)), or there was a statistically significant difference, but only in the long-term (18 months and overall survival, such as in the BTSG trial 6901 (Walker et al, 1978) or in the RTOG 7401-ECOG 1374 (Change et al, 1983; Nelson et al, 1988)). A potential explanation of the marginally significant results may have been the relatively small number of patients in some of the trials. To have an 80% probability of detecting a 25% increase in median survival at the 5% significance level, one would need a clinical trial following 250 patients per arm until death. The BTSG trials which were among the largest multi-institution studies had fewer than 150 patients in each treatment arm. Most single-institution studies have had fewer than 50 patients per treatment arm. Using the results from 16 randomized clinical trials involving more than 3000 patients, Fine et al (1993) performed a meta-analysis of radiation therapy with and without adjuvant chemotherapy for malignant gliomas in adults. The estimated increase in survival for patients treated with combination radiation and chemotherapy was 10.1% at 1 year and 8.6% at 2 years. This absolute increase in survival in patients treated with chemotherapy represents relative increases of 23.4% at 1 year survival and 52.4% at 2 years' survival. When the prognostic variables of age and histology were incorporated in the analysis, the data suggested that the survival benefit from chemotherapy appeared earlier in patients with anaplastic astrocytoma than in patients with glioblastoma multiforme: the maximal survival advantage was seen at 12–18 months for patients with anaplastic astrocytoma versus 18–24 months for patients with glioblastoma. There were methodological problems associated with this meta-analysis, the most important being the use of reported clinical trial results, some of which were incomplete, rather than individual patient data to perform the analysis.

The route of chemotherapy administration was assessed in the BTSG trial 8301 (Shapiro et al, 1992). Patients with newly resected malignant glioma received either intra-carotid BCNU or intravenous BCNU with or without intravenous 5-fluorouracil (5-FU). All patients also received radiation therapy. Intravenous BCNU was significantly better than intra-arterial BCNU ($P = 0.03$), mainly because of the serious toxicity that was observed in the intra-arterial group; 16 patients (9.5%) developed irreversible encephalopathy with CT evidence of cerebral oedema, and 26 patients (15.5%) developed visual loss ipsilateral to the infused carotid artery. Administration of 5-FU did not influence survival. The survival rate between GBM patients treated with intravenous vs intra-arterial BCNU did not differ, but it was worse for patients with anaplastic astrocytoma treated with intra-arterial BCNU than for those receiving intravenous BCNU ($P = 0.009$). Similarly, a phase II SWOG study of intra-arterial administration of cisplatin concomitant with or before radiation therapy led to thromboembolic problems in 14% of the courses. Five of 27 patients died prior to treatment completion (Mortimer et al, 1992).

The value of adjuvant chemotherapy varies according to age and tumour grade. Long-term follow-up of the RTOG 7401-ECOG 1374 trial (18) showed that for patients aged 40–60 years there was statistically significant increase in overall survival when BCNU was added to 6000 cGy ($P < 0.01$) with an increase in 2-year survival from 8 to 23%. For patients older than 60 years of age, the addition of chemotherapy to radiation therapy did not improve survival. In the 40–60 year age group, the beneficial effect of BCNU was apparent in both histological groups (anaplastic astrocytoma and glioblastoma multiforme). Although few confirmatory autopsies were available, long-term survival in patients with anaplastic astrocytomas who were treated with 6000 cGy + BCNU suggested no significant additional CNS toxicity compared to 6000 cGy alone (Nelson et al, 1988).

While designed to compare the efficacy of different treatment modalities in the adjuvant setting, multiple prospective randomized trials have also identified association among specific prognostic variables and the survival time for these patients. These include histologic grade and type, age and performance score (Karnofsky or ECOG). Glioblastoma multiforme patients survive approximately half as long as those harbouring anaplastic astrocytoma. Patients older than 60 have a significantly shorter survival regardless of the grade of tumour. Patients with Karnofsky score above 90 have one-third the death rate of patients with ratings of 40 or below (Walker et al, 1980; Germain and Margulies 1993). Some investigators have also reported that extent of tumour resection as estimated by the neurosurgeon provides prognostic information. Patients who undergo gross total tumour resection fare better than those with subtotal resection or biopsy alone. The improvement may be explained partially by tumour size and location; that is, patients with smaller tumours in non-eloquent brain may fare better, regardless of surgical procedure.

The value of neoadjuvant chemotherapy administered prior to surgery or prior to radiation therapy in high-grade gliomas remains an open question. Radiation-induced changes to the integrity of the blood–brain barrier make it very difficult to evaluate the efficacy of the chemotherapy administered during or shortly after radiation therapy even in patients with measurable disease. Neoadjuvant chemotherapy has the potential to greatly enhance the discovery of truly active agents as well as the early identification of ineffective therapies in a disease where chemotherapy has been only marginally effective. In a phase II setting, Grossman et al administered BCNU and CDDP as a 72-h continuous intravenous infusion

*© 2000 Cancer Research Campaign*

**Table 2** Selected chemotherapy trials for recurrent and progressive astrocytomas

| Agent | Total patients | Pretreated patients (%) | AA/GBM (%) | Response (%) | Median survival (weeks) | Reference |
|---|---|---|---|---|---|---|
| Nitrosourea regimens | | | | | | |
| BCNU | 91 | – | – | 31 | 16 | Broder and Carter, 1970 |
| CCNU | 116 | – | – | 30 | 22 | Hoogstraten et al, 1973 |
| BCNU + AZQ | 42 | 50 | 24/76 | 21GB, 30AA | 23 | Yung et al, 1989 |
| BCNU + PCBZ | 45 | – | – | 29 | – | Gutin et al, 1975 |
| BCNU + IFN-α | 35 | 0 | – | 26 | 52 | Buckner et al, 1989 |
| CCNU + procarbazine + vincristine | 46 | 58 | 26/26 | 29 | – | Levin et al, 1980 |
| PCBZ-containing regimens | | | | | | |
| PCBZ | 99 | 96 | 37/46 | 28 | – | Rodriguez et al, 1989 |
| PCBZ + VCR + Thiotepa | 20 | 100 | 20/80 | 15 | 52 | Ameri et al, 1993 |
| MOP | 27 | 29 | 22/62 | 52 | 20GB, 81AA | Coyle et al, 1990 |
| Platinum-containing regimens | | | | | | |
| CDDP | 45 | 100 | – | 17 | 20 | Spence et al, 1992 |
| CBDCA | 34 | 100 | 50/50 | 11 | 24 | Yung et al, 1991 |
| CDDP + VP16 | 33 | 100 | 31/69 | 17 | 24 | Buckner et al, 1990 |
| CBDCA + VP16 | 38 | 100 | 21/79 | 21 | 47.5 | Jeremic et al, 1992 |
| CBDCA + VP16 + Ifosfamide | 36 | 100 | 22/72 | 28 | 29 | Sanson et al, 1996 |
| Other regimens | | | | | | |
| AZQ | 68 | 38 | 32/68 | 26 | 22 | EORTC, 1985 |
| Melphalan | 30 | 100 | 46/54 | 3 | 8 | Chamberlain et al, 1988 |
| Ifosfamide | 16 | 100 | 50/50 | 0 | 19 | Elliott et al, 1991 |
| VM26 | 20 | 100 | – | 20 | 24 | Kessinger et al, 1979 |
| VP16 | 35 | 100 | 54/46 | 17 | 18 | Tirelli et al, 1984 |
| Fludarabine | 15 | 53 | 45/55 | 3 | 19 | Cascino et al, 1988 |
| Paclitaxel | 20 | 100 | 40/40 | 20 | – | Chamberlain and Kormanik, 1995 |

BCNU = carmustine, CCNU = lomustine, PCBZ = procarbazine, AZQ = aziridinylbenzoquinone, CDDP = cisplatin, CBDCA = carboplatin, NP26 = teniposide, VP16 = etoposide, IFN-α = interferon-alpha, MOP = mechlorethamine, vincristine, procarbazine, VCR = vincristine.

prior to radiation therapy (Grossman et al, 1997). Of the 52 patients studied, 42% had a partial response and 53% had stable disease. Only two patients (4%) developed progressive disease while on treatment. The 1- and 3-year survival rates were 64% and 12% respectively. Similar results were reported by Gilbert et al (1993).

In order to address this question definitively, two current intergroup phase III trials (NCCTG/SWOG 93–72–52 and ECOG/SWOG 2394) compare BCNU + CDDP at different doses and schedules either prior to or concomitantly with radiation. Both trials will assess the effect of chemotherapy administered prior to radiation therapy in glioblastoma patients. The NCCTG/SWOG study completed accrual in June 1999 and the first analysis of the data is anticipated by the end of the year 2000.

In summary, chemotherapy has a limited role in the management of newly diagnosed patients with high-grade astrocytomas. Nitrosourea-containing regimens administered with and after radiation therapy appear to produce modest survival advantage in younger (< 60 years of age) GBM patients in some but not all studies and in patients with anaplastic astrocytomas. Intra-arterial administration of BCNU and cisplatin appears to result in unacceptable toxicity. The effectiveness of combination regimens containing both cisplatin and nitrosoureas and the value of neo-adjuvant chemotherapy is a matter of active investigation.

### CHEMOTHERAPY FOR HIGH-GRADE OLIGODENDROGLIOMAS AND OLIGOASTROCYTOMAS

In the recent years, anaplastic oligodendrogliomas have been identified as particularly sensitive to chemotherapy, as compared to astrocytomas. Cairncross and Macdonald in 1988 reported on use of nitrosourea-based chemotherapy mainly PCV for treatment of recurrent anaplastic oligodendroglioma (Cairncross and Macdonald, 1988, 1991; Cairncross et al, 1992). In a small series of eight patients, all patients responded to chemotherapy including two with systemic metastasis. The median duration of response in these previously irradiated patients was 15 months (range 8–36 months). The same authors reported responses to PCV administered before radiation therapy in patients with newly diagnosed aggressive oligodendrogliomas and measurable disease after initial surgery (Macdonald et al, 1990). These studies led to a multicentre phase II study in which patients with measurable newly diagnosed or recurrent contrast-enhancing anaplastic oligodendrogliomas were treated with up to six cycles of PCV (Cairncross et al, 1994). Of 24 eligible patients, 18 (75%) responded, nine completely (38%), four had stable disease and two progressed during the first cycle of PCV. Previously irradiated patients were as likely to respond to PCV as newly diagnosed (11 of 15 (75%) versus 7 of 9 (78%)). The median time to progression was at least 25.2 months for the complete responders, 14.2 months for partial responders, and 6.8 months for stable patients.

The role of adjuvant chemotherapy for patients with oligodendroglioma remains uncertain. Based on promising results in patients with either newly diagnosed or recurrent disease, an intergroup study (RTOG 9402) is in progress comparing radiation alone vs four cycles of PCV followed by radiation in patients with anaplastic oligodendroglioma/oligoastrocytoma.

In addition to PCV, other chemotherapy agents are active against oligodendrogliomas such as melphalan (Green et al, 1983;

 *© 2000 Cancer Research Campaign*

Brown et al, 1990), BCNU and AZQ (Cairncross and Macdonald, 1988). Data examining the role of these agents as salvage chemotherapy in patients who have failed PCV are limited. While PCV has been proven to have activity in patients who have previously responded to AZQ or received adjuvant BCNU therapy, the opposite does not always hold true. AZQ and cisplatin were ineffective in four patients who relapsed after PCV, while two patients who had previously responded to PCV had brief partial responses after intravenous treatment with melphalan (Brown et al, 1990). As a result, there is no standard second-line treatment for patients with recurrent disease who have failed PCV.

Anaplastic oligoastrocytoma patients are usually treated similarly to those with pure oligodendroglioma. Glass et al (1992) and Kyritsis et al (1993) have reported that oligoastrocytomas also respond predictably to PCV with objective response rates of 64% and 42%, respectively, in newly diagnosed tumours. Similarly, oligoastrocytoma patients treated with PCV at first relapse had a median time from recurrence to death of 5.6 years, while the median relapse-free survival was 1.3 years (Kyritsis et al, 1993).

### CHEMOTHERAPY FOR RECURRENT HIGH-GRADE ASTROCYTOMAS

Different agents and combinations have been employed in phase II trials for treatment of recurrent and progressive supratentorial astrocytomas (Table 2), but results have been quite disappointing. Response rates are low and time to progression is short, varying from a few to several months. Nitrosoureas have the most consistent activity with response rates ranging from 21 to 31% (Table 2). The overwhelming majority of chemotherapy trials for patients with recurrent high-grade glioma include both patients with anaplastic astrocytoma and glioblastoma multiforme. Of note, some of the studies referenced in Table 2 were conducted in the pre-CT era and the results should be viewed with caution since response was often assessed based on clinical criteria only. In addition, results reported regarding paclitaxel as summarized in Table 2 may represent an underestimation of the actual drug activity since doses employed were subtherapeutic for patients on anticonvulsants. The same applies for topoisomerase-I inhibitors such as 9-AC (Grossman et al, 1998). Encouraging results were also reported with the use of MOP combination (mechlorethamine, vincristine, procarbazine) in recurrent malignant glioma patients, including a response rate of 37% in glioblastomas and 100% in anaplastic astrocytoma (Coyle et al, 1990). Unfortunately, these results could not be reproduced in a recent larger NCCTG study (Galanis et al, 1998). Although phase III comparison data are not available, as Table 2 indicates, there is no consistent evidence that any combination is superior to single-agent nitrosourea chemotherapy for patients with recurrent gliomas.

Given the increasingly widespread use of nitrosoureas in the adjuvant setting for both anaplastic astrocytomas and glioblastoma multiforme, options at the time of relapse are even more limited. Consideration could be given to the use of procarbazine in patients who failed BCNU in the adjuvant setting. In one series of 99 high-grade glioma patients (96 of whom were pretreated), single-agent procarbazine resulted in a response rate of 28% (Rodriguez et al, 1989). Platinum compounds may also offer limited benefit with response rates ranging from 11 to 21% (Buckner et al, 1990; Yung et al, 1991; Jeremic et al, 1992; Spence et al, 1992). More recently, interesting results were obtained with the ICE regimen (ifosfamide, carboplatin and etoposide) in a series of 36 well-defined patients who were all pretreated with surgery, irradiation and nitrosourea-based chemotherapy. Five complete responses (CR) and five partial responses (PR) were reported with an overall response rate of 28%. Unfortunately, median survival (29 weeks) for all patients remained low despite a median survival of 44 weeks for the responders, and it was achieved at a price of severe haematologic toxicity (Sanson et al, 1996).

Dacarbazine is another agent that has been reported to have activity against recurrent glioma. However, a recently completed NCCTG study indicated a response rate of less than 5% when dacarbazine was employed in patients with recurrent gliomas (unpublished data). There has also been considerable interest in temozolomide, an imidotetrazine analogue of dacarbazine (DTIC). Unlike dacarbazine, temozolomide does not require hepatic activation, and it displays excellent oral bioavailability (Newlands et al, 1997). Based on encouraging responses observed during phase I studies (Newlands et al, 1992), temozolomide was administered to 48 patients with recurrent glioma after radiotherapy. Objective clinical radiologic responses were reported in 25% of the patients (Newlands et al, 1996). In another multicentre phase II study in patients with progressive or recurrent supratentorial high-grade gliomas, 11% of the patients achieved objective response with a median duration of 4.6 months and another 47% had stable disease (Bower et al, 1997). In a phase III study comparing temozolomide with procarbazine in patients who have failed nitrosourea-based chemotherapy for recurrent glioblastoma multiforme, temozolomide led to a response rate of 4.7% and a response duration of 85 days as compared to a response rate of 3% for procarbazine and a response duration of 99 days. There was a statistically significant difference in progression-free survival favouring the temozolomide arm. However, there was no significant difference in median or overall survival. Preliminary quality-of-life analysis data seemed to indicate that quality-of-life until progression was better in the temozolomide arm (Yung et al, 1999). In a phase II trial of 162 patients with recurrent anaplastic astrocytomas, temozolomide led to a response rate of 35%, while 27% of the patients had stable disease (Prados et al, 1999). The 6- and 12-month progression-free survival was 46% and 24% respectively. The response was highest for patients who had not received prior chemotherapy (39%) and lower for patients who received prior nitrosoureas with or without procarbazine (22%). Temozolomide has been approved for patients with recurrent GBM in Europe and with recurrent anaplastic astrocytoma in the US, the latter approval being contingent upon confirmatory data. Combination studies with cis-retinoic acid, inter-feron alpha nitrosoureas (BCNU) and radiation are underway (Levin, 2000; Newlands 1997*a*; Newlands 1997*b*). Finally, ongoing trials will address the role of temozolomide as a single agent or in combination with BCNU for newly diagnosed anaplastic astrocytoma patients.

Recently published results also indicate that the topoisomerase I inhibitor CPT-11 does have activity in patients with recurrent disease. In a study of 60 patients, 48% of whom had received prior nitrosourea-based chemotherapy, a 15% partial response rate was observed. The median survival was 42 weeks for the 48 patients with GBM and not reached yet (more than 40 weeks) for the ten patients with anaplastic astrocytoma. The low incidence of severe toxicity and low plasma concentration of CPT-11 and its metabolite SN-38 suggested that concurrent treatment within anticonvulsants or dexamethasone may have enhanced drug clearance (Friedman et al, 1999).

Despite the large number of phase II studies and continued use of chemotherapy in patients with recurrent high-grade glioma,

© 2000 Cancer Research Campaign information concerning functional efficacy of chemotherapy in this setting is not easy to find. This may be due either to the difficulty of measuring quality of life and functional status in brain tumour patients or simply to the absence of planned evaluation of these parameters as a measure of efficacy. With new quality of life scales (Osaba et al, 1996) and functional indices (Grant et al, 1994) this should no longer be an obstacle.

Controlled release of biodegradable polymers also provides a novel approach. Two polyanhydride systems have been studied extensively; p(CPP-SA) for hydrophobic drugs such as BCNU; p(FAP-SA) for hydrophilic compounds, and those susceptible to hydrolysis such as methotrexate and cisplatin (Brem et al, 1992; Domb et al, 1991). Brem et al (1992) have established the safety of this methodology. In a recent randomized placebo-controlled trial of 222 patients with recurrent gliomas, 6-month survival was greater in glioblastoma patients treated with BCNU wafers than in the placebo group (64% vs 44%) (Brem et al, 1995). Despite a trend favouring the BCNU implant group, the differences in median and overall survival did not reach statistical significance. In a multiple regression analysis model, carmustine polymer showed a significant beneficial effect in recurrent glioblastoma patients (hazard ratio = 0.67, $P = 0.02$) (Brem et al, 1995). Future studies employing this technology will examine the utility of BCNU-polymer therapy in newly diagnosed patients, the efficacy and toxicity of escalating doses of BCNU, and the effects of other cytotoxic drugs delivered in this fashion.

Autologous bone marrow transplantation (ABMT) has had few practitioners generally because of the low response rate of high-grade gliomas to any form of chemotherapy. Use of high-dose BCNU (800 $mg/m^2$ to 1400 $mg/m^2$) resulted in responses in 33–44% of the patients and treatment-related deaths in 17–32% of the patients mainly because of infection complications, interstitial pneumonitis and drug-induced severe hepatitis (Phillips et al, 1986). Overall, none of those studies have shown a significant benefit as compared to conventional dose of nitrosourea therapy (Phillips et al, 1986; Giannone and Wolff, 1987; Fine and Antman, 1992). Similarly, the use of high-dose etoposide in the setting of ABMT (1000–2400 $mg/m^2$) resulted in a response rate of 19% and a toxic death rate of 15% (Giannone and Wolff, 1987).

In summary, nitrosourea-based regimens can be considered as standard first-line treatment for patients with recurrent high-grade gliomas who have not received prior nitrosoureas. The superiority of combination regimens, of nitrosourea-based, to single-agent nitrosoureas in this setting has not been proven. For patients who have failed prior nitrosoureas, chemotherapy approaches include temozolomide, procarbazine, platinum compounds and possibly CPT-11. Given the limited efficacy of all available treatments for recurrent high-grade gliomas, participation in phase II or III clinical trials should be encouraged.

### FUTURE DIRECTIONS

The lack of effective curative treatments for most high-grade glioma patients has led to several efforts for development of new therapeutic approaches. In recent years, explosive development of molecular biology techniques has allowed the molecular characterization of high-grade gliomas. In addition, the role of pharmacologic approaches employing small molecules as well as gene transfer techniques aiming at specific molecular targets is currently under assessment in clinical trials. It is the authors' hope that, in the future, molecular/genetic characterization of high-grade gliomas will allow use of individualized rational treatments depending on the tumour's genetic make-up. In addition, emerging data on the role of angiogenesis in tumour invasion and the challenge of the dogma that CNS represents an immunologic sanctuary may lead to new approaches in the treatment of high-grade gliomas. In this process, a lot remains to be learned about interactions and integration of these newer approaches with traditional methods used in the treatment of high-grade gliomas such as surgery, radiation therapy and conventional chemotherapy regimens.

### Chemotherapeutic agents

The explosive development of small molecules targeting cell cycle signalling opens new directions in the treatment of high-grade gliomas. Figures 1 and 2 describe cytogenetic changes as well as cell cycle regulators with possible pathogenetic importance in the development of high-grade malignant gliomas. Among these agents currently in preclinical or early stages of clinical development are rapamycin analogues that inhibit the action of protein kinase m-TOR or FRAP (Beretta et al, 1996), farnesyl transferase inhibitors (Kohl et al, 1993), and tyrosine kinase inhibitors such as tyrphostins (Yoneda et al, 1991; Siu et al, 1999; Karp et al, 1999; Yung et al, 1999). Tyrosine kinases may play an important role in

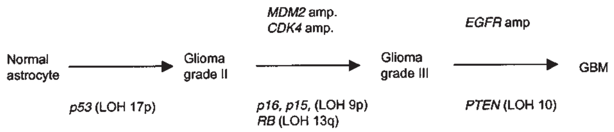

**Figure 1** Gene alterations associated with the development of malignant gliomas. (Amp = amplification; LOH = loss of heterozygosity.) Reproduced with permission from Galanis E and James CD (1999) Brain Tumors In: *Molecular Biology in Cancer Medicine, 2nd Edn,* Kurzrock R and Talpaz M (ed.)

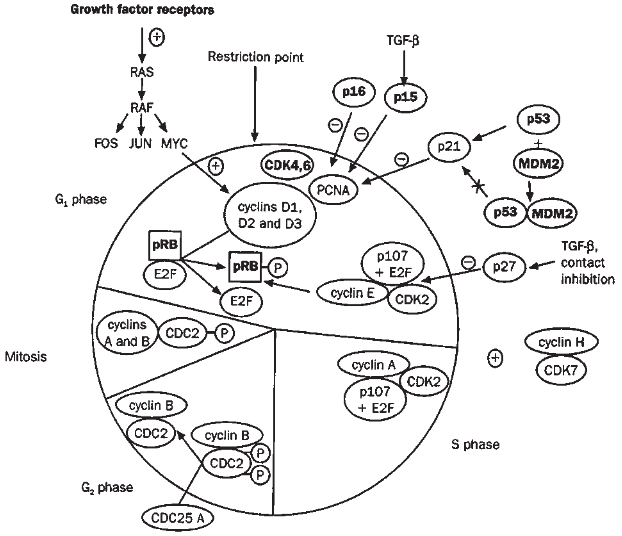

**Figure 2** Cell cycle regulators. Positive (+) and negative(-) regulators of cyclin/CDK are indicated. Protein whose genes are altered in brain tumors are shown in bold. (CDK = cyclin dependent kinase; TGF = transforming growth factor, RB = retinoblastoma) (Reproduced with permission from Galanis E and James CD (1999) Brain Tumors In: *Molecular Biology in Cancer Medicine, 2nd Edn*, Kurzrock R and Talpaz M, (ed.)

 *© 2000 Cancer Research Campaign*

pathogenesis of malignant gliomas. For example, epidermal growth factor receptor, an oncogene amplified in approximately 50% of glioblastomas, has tyrosine kinase activity. Other approaches targeting EGFR include monoclonal antibodies and antisense oligonucleotides with EGFR mRNA being a potential target (80).

Another promising category of new small molecule pharmaceuticals include anti-invasion molecules. Matrix metalloproteinases are proteases overexpressed in tumours (Lampert et al, 1998) that degrade macromolecules of the extracellular matrix and are responsible for tumour invasion. Matrix metalloproteinase inhibitors (MMPIs) such as marimastat are currently in early stages of clinical development while other anti-invasion molecules such as quinazoline derivatives (they inhibit glioblastoma cell adhesion and activation and induce apoptosis in vitro) are undergoing preclinical testing (Krishna et al, 1998).

Phase II trials are also underway, trying to assess the effect of the combination of $O^6$-benzylguanine plus BCNU in patients with recurrent high-grade gliomas. $O^6$-benzylguanine inhibits $O^6$-alkylguanine-DNA alkyltransferase (AGT), a repair protein that appears to predict resistance to nitrosoureas and other related alkylators (Belanich et al, 1996; Dolan and Pegg, 1997).

Finally, the role of newer agents that increase the permeability of blood–brain barrier to hydrophilic chemotherapy agents is currently under investigation. The bradykinin analogue RMP-7 has been administered intravenously (Ford et al, 1998) or intraarterially (Cloughesy et al, 1999) in combination with carboplatin and it is currently undergoing evaluation in phase II and III studies.

### Angiogenesis inhibition

Glial tumours are characteristically hypervascular as endothelial proliferation is a hallmark pathologic feature of high-grade glioma (Burger et al, 1991). This neovascularization typically produces a striking pattern of capillary tufts resembling renal glomeruli which are found throughout the tumour (Russell and Rubinstein, 1989). Glioma vasculature is usually abnormal, both from a structural as well as a functional standpoint (Luse 1960; Kaye and Laws, 1995). The presumed causal link between angiogenesis and prognosis via metastasis which exists for extracranial tumours may or may not be applicable to gliomas and other mechanisms by which angiogenesis may directly affect the clinical course of gliomas must be postulated. Foremost among these is the likelihood that aggressive local growth is facilitated by intense angiogenesis. The issue of the prognostic significance of angiogenesis in glial neoplasm is complex and in need of further study as indicated by conflicting reports regarding its prognostic significance and further complicated by the fact that angiogenesis is by definition associated with increasing tumour grade and poor prognosis (Burger et al, 1985; Cohadon et al, 1985; Fulling and Gercia, 1985; Daums-Duportet al, 1988). A number of factors have been implicated in glioma angiogenesis including vascular endothelial growth factor (VEGF) (Plate et al, 1994), fibroblast growth factor (FGF) (Li et al, 1994), epidermal growth factor (EGF) (Folkman and Klagsbrun, 1987), transforming growth factors (TFG) (Folkman and Klagsbrun, 1987) and platelet-derived growth factor (PDGF) (Folkman and Klagsbrun, 1987).

Anti-angiogenesis therapy for brain tumours offers several additional advantages as well as significant limitations not found in extracranial neoplasms. The main advantage is that gliomas generally do not metastasize. Therapy targeted to local disease could theoretically be effective. Also, the adult brain contains few dividing cells (except perhaps endothelial cells themselves) and does not under routine circumstances express normal angiogenesis (Russell and Rubinstein, 1989).

Anti-angiogenesis therapy directed at highly vascular gliomas might therefore be expected to be less toxic to the surrounding brain. In addition, with sensitive modern imaging studies, tumour growth and response to therapy can be followed non-invasively. Agents with anti-angiogenesis potential currently or recently in clinical trials include SU101 that blocks the angiogenetic function of PDGF as well as VEGF by inhibiting a receptor tyrosine kinase. A phase III study currently ongoing in the USA and Canada compares SU101 with procarbazine in patients with recurrent gliomas. Other agents currently in clinical trials are the fumagillin analogue TNP-470 that appears to have very specific effects on endothelial cell growth, producing cytostatic growth arrest (Takamiya et al, 1994), and thalidomide (D’Amato et al, 1994). A recently completed trial of thalidomide in patients with recurrent high-grade gliomas, showed minimal antitumor activity (PR = 6%), although the drug was generally well tolerated (Fine 2000). Finally, other potential agents include angiogenesis inhibitors, angiostatin and endostatin (O’Reilly 1997; O’Reilly et al, 1997).

### Gene therapy

Because intracranial recurrence rather than systemic metastases characterizes the pattern of relapse in gliomas and brain tumours, they became one of the first targets for human gene therapy for cancer. Gene therapy research has evolved from the initial retroviral vectors to herpes virus vectors, adenoviral vectors and HSV (herpes simplex virus)/AAV (adeno-associated virus) hybrid vectors, in order to improve transduction rates, transgene capacity and eliminate the possibility of neurotoxicity. Patients have been treated by stereotactic intratumoural injections of fibroblasts producing retroviral vectors that deliver the gene encoding the enzyme HSV thymidine kinase with subsequent systemic ganciclovir treatment (Oldfield et al, 1993). Fifteen patients with primary or metastatic brain tumours were entered in the first study. MRI suggested regression in the volume of the gadolinium-enhancing portion of the tumours in the early period after treatment in eight patients. In four patients the enhanced tumour portion was not detected for periods of 4–18 months. Despite the low transduction efficiency (17%), bystander effect resulted in additional killing of the tumour cells (Oldfield et al, 1993). In order to give the patients the additional benefit of tumour debulking, 30 patients with recurrent GBM had tumour resection followed by cavitary injection of the vector producer cell line (GLI 328) and gancyclovir treatment. Five patients were alive at 17–32 months after resection (Berger et al, 1997). A phase III trial of GLI 328 in patients with newly diagnosed glioblastoma was recently completed in multiple centres worldwide. Additional gene delivery systems lately introduced in clinical trials for the treatment of recurrent gliomas are an HSV-tk adenovirus and a replication defective p53 adenovirus (an approach justified based on the high p53 mutation rate in high-grade gliomas, ranging between 25 and 50%). The fact that CNS gliomas rarely metastasize makes them a good target for local delivery achieved through gene transfer approaches; however, improvement of the available gene delivery systems may be necessary to realize the full potential of this treatment approach. Despite the presence of connexin mediated gap junctions that facilitate the ‘bystander’ effect, the diffuse invasion of the brain by high-grade glioma cells beyond imaging

*© 2000 Cancer Research Campaign*

abnormalities makes the efficiency of the available gene transfer vectors problematic even when administered after optimal surgical debulking. A possible answer to this question could be replication competent viruses. Along this line, a phase I clinical trial has been recently completed, employing a double mutant herpes simplex virus vector that selectively replicates in glioma cells but not normal brain (Markert et al, 1999; Mineta et al, 1995). In addition, ONYX-015, an E1B attenuated adenovirus that can selectively replicate in cells with malfunctioning p53 (Bischoff et al, 1996).

### Immunotherapy

The specifics of the immune response against gliomas are not very well understood (Dietrich et al, 1997); nevertheless, more information has been accumulating regarding the role of immunosuppressive cytokines such as TGF-β2 or interleukin (IL)-10 and Fas ligand expression by glioma cells and remain to be translated into useful therapeutic approaches, potentially after further development of efficient gene transfer techniques. Immunotherapy approaches tested to date include administration of IL-2 with LAK or tumour infiltrating lymphocytes (TIL cells). Despite the encouraging results in animal models, human trials were met with little success (Yoshida et al, 1990; Barba et al, 1989). In another approach, radiolabelled anti-EGF receptor antibodies have been injected into the tumour bed following resection of recurrent gliomas. The dosimetry and toxicity of this approach have been reported (Brady et al, 1992). Efficacy studies are ongoing.

A different monoclonal antibody approach involves the injection of a I131 labelled anti-tenascin monoclonal antibody into the resection cavities of recurrent or newly diagnosed glioma patients (Cokgor et al, 1999). Phase II trials are ongoing. Finally, the therapeutic potential of immunotoxins such as IL-4 bound pseudomonas exotoxin A is under investigation. The latter approach is based on the observation that 60–80% of high grade gliomas express IL-4 receptor as compared to virtually no expression of the same receptor in normal brain (Weber et al, 1999).

In summary, nitrosourea-based chemotherapy in the adjuvant setting has limited value in the management of patients with GBM and its use should be individualized. Development of new drugs should follow strict methodologic rules and take into consideration other outcome measures such as quality of life. Anaplastic astrocytomas are more chemosensitive than GBMs. Use of a nitrosourea-based regimen in the adjuvant setting appears to be associated with a survival benefit for anaplastic astrocytoma patients. For patients with recurrent high-grade astrocytomas, options include nitrosoureas, especially for previously untreated patients, the imidotetrazine analogue, temozolomide, platinum-based regimens and procarbazine. Participation in clinical trials should be particularly encouraged in this setting. Oligodendrogliomas are chemosensitive tumours. PCV is indicated in relapse and current studies will define its role in the adjuvant setting. Innovative strategies including small molecule pharmaceuticals, anti-invasion agents, anti-angiogenesis agents and gene therapy approaches and their interactions with 'classic' chemotherapy agents need to be further investigated.

### REFERENCES

Ameri A, Poisson M, Chen QM, et al (1993) Treatment of recurrent malignant supratentorial gliomas with the association of procarbazine, thiotepa and vincristine: a phase II study. *J Neuro-Oncol* **17**: 43–46

Barba D, Saris SC, Holder C, et al (1989) Intratumoral LAK cell and interleukin-2 therapy of human gliomas. *J Neurosurg* **70**: 175

Belanich M, Pastor M, Randall T, et al (1996) Retrospective study of the correlation between the DNA repair protein alkyltransferase and survival of brain tumor patients treated with carmustine. *Cancer Res* **56**: 783–788

Beretta L, Gingras A-C, Svitkin YV, Hall MN and Sonenberg N (1996) Rapamycin blocks the phosphorylation of 4E-BP1 and inhibits cap-dependent initiation of translation. *Embo J* **15**: 6578–6664

Berger MS, Prados MD, Van Gilder JC, et al (1997) Phase II Trial of GLI 328 HSV-TK gene therapy in recurrent glioblastoma. *Cancer Gene Ther* **4**: 542

Bischoff JR, Kirn DH, Williams A, Heise C, Horn S, Muna M, et al (1996) An adenovirus mutant that replicates selectively in p53-deficient human tumors cells. *Science* **274**: 373–376

Bleehen NM, Roberts JT, Senanayake F, et al (1998) Medical Research Council (MRC) randomised trial of adjuvant chemotherapy in high grade glioma (HGG)-BR05. *Proc Am Soc Clin Oncol* **17**: 400a

Bower M, Newlands ES, Bleehen NM, et al (1997) Multicentre CRC phase II trial of temozolomide in recurrent or progressive high-grade glioma. *Cancer Chemother Pharmacol* **40**: 484–488

Brada M and Sharpe G (1996) Chemotherapy of high-grade gliomas: beginning of a new era or the end of the old? *Eur J Cancer* **32A**: 2193–2194

Brady LW, Miyamoto C, Woo DV, et al (1992) Malignant astrocytomas treated with iodine-125 labeled monoclonal antibody 425 against epidermal growth factor receptor: a phase II trial. *Int J Radiat Oncol Biol Phys* **22**: 225

Brem H, Domb A, Lenartz D, et al (1992) Brain biocompatibility of a biodegradable controlled release polymer consisting of anhydride copolymer of fatty acid and sebacic acid. *J Control Rel* **19**: 325–330

Brem H, Piantadosi S, Burger PC, et al (1995) Placebo-controlled trial of safety and efficacy of intraoperative controlled delivery by biodegradable polymers of chemotherapy for recurrent gliomas. The Polymer–Brain Tumor Treatment Group. *Lancet* **345**: 1008–1012

Broder LE and Carter SK (1970) *Clinical brochure BCNU (NSC-409962)*. Bethesda, MD

Brown M, Cairncross JG, Vick NA, et al (1990) Differential response of recurrent oligodendrogliomas versus astrocytomas to intravenous melphalan. *Neurology* **40**: 397–398

Buckner JC, Brown LD and Cascino TL (1989) Recombinant alpha-interferon and BCNU in recurrent gliomas. *J Biol Response Mod* **8**: 332–333

Buckner JC, Brown LD and Cascino TL (1990) Phase II evaluation of infusional etoposide and cisplatin in patients with recurrent astrocytoma. *J Neuro-Oncol* **9**: 249–254

Burger P, Vogel F, Green S and Strike T (1985) Glioblastoma multiforme and anaplastic astrocytoma; pathologic criteria and prognostic implications. *Cancer* **56**: 1106–1111

Burger P, Scheithauer B and Vogel F (1991) *Surgical Pathology of the Nervous System and its Coverings*. Churchill Livingstone: New York

Cairncross JG and Macdonald DR (1988) Successful chemotherapy for recurrent malignant oligodendroglioma. *Ann Neurol* **23**: 360–364

Cairncross JG and MacDonald DR (1991) Chemotherapy for oligodendroglioma: progress report. *Arch Neurol* **48**: 225

Cairncross JG, Macdonald DR and Ramsay DA (1992) Aggressive oligodendroglioma: a chemosensitive tumor. *Neurosurgery* **31**: 78–82

Cairncross G, Macdonald D, Ludwin S, et al (1994) Chemotherapy for anaplastic oligodendroglioma. *J Clin Oncol* **12**: 2013–2021

Cascino TL, Brown LD and Morton FM (1988) Evaluation of fludarabine phosphate in patients with recurrent glioma. *Am J Clin Oncol* **11**: 586–588

Chamberlain MC and Kormanik P (1995) Salvage chemotherapy with paclitaxel for recurrent primary brain tumors. *J Clin Oncol* **13**: 2066–2071

Chamberlain MC, Prados MD and Silver PA (1988) A phase II trial of oral melphalan in recurrent primary brain tumors. *Am J Clin Oncol* **11**: 52–54

Chang CH, Horton J, Schoenfeld D, et al (1983) Comparison of postoperative radiotherapy and combined postoperative radiotherapy and chemotherapy in the multidisciplinary management of malignant gliomas. *Cancer* **52**: 997

Chang SM, Kuhn JG, Rizzo J, et al (1998) Phase I study of paclitaxel in patients with recurrent malignant glioma: a North American brain tumor consortium report. *J Clin Oncol* **16**: 2188–2194

Cloughesy TF, Blaack KL, Gobin UP, et al (1999) Intra-arterial Cereport (RMP-7) and carboplatin: a dose escalation study for recurrent malignant gliomas. *Neurosurg* **44**: 270–278

Cohadon F, Aouad N, Rougier A, Vital C, Rivel J and Dartigues J (1985) Histologic and nonhistologic factors correlated with survival time in supratentorial astrocytic tumors. *J Neuro-Oncol* **3**: 105–111

Cokgor I, Akabani G, Friedman AH (1999) Newly diagnosed malignant glioma patients treated with 131I/anti-tenascin monoclonal antibody (MAB) 81C6 via

*© 2000 Cancer Research Campaign*

surgically created resection cavities: Results of a phase I trial. *Neuro-Oncol* **1**: S107
Coyle T, Baptista J and Winfield J (1990) Mechlorethamine, vincristine and procarbazine chemotherapy for recurrent high-grade glioma in adults: a phase II study. *J Clin Oncol* **8**: 2014–2018
D'Amato RJ, Loughnan MS, Flynn E and Folkman J (1994) Thalidomide is an inhibitor of angiogenesis. *Proc Natl Acad Sci USA* **91**: 4082–4085
Daums-Duport C, Scheithauer B, O'Fallon J and Kelly P (1988) Grading of astrocytomas: a simple and reproducible method. *Cancer* **62**: 2152–2165
Deutsch M, Green SB, Strike TA, et al (1989) Results of a randomized trial comparing BCNU plus radiotherapy, streptozotocin plus radiotherapy, BCNU plus hyperfractionated radiotherapy, and BCNU following misonidazole plus radiotherapy in the postoperative treatment of malignant glioma. *Int J Radiat Oncol Biol Phys* **16**: 1389
Dietrich PY, Saas P, Walker PR, et al (1997) Immunobiology of gliomas: new perspectives for therapy. *Ann NY Acad Sci* **824**: 124–440
Dinapoli RP, Brown LD, Arusell RM, et al (1993) Phase III comparative evaluation of PCNU and carmustine combined with radiation therapy for high grade glioma. *J Clin Oncol* **11**: 1316
Dolan ME and Pegg AE (1997) $O^6$-benzylguanine and its role in chemotherapy. *Clin Cancer Res* **3**: 837–847
Domb A, Bogdansky S, Olivi A, et al (1991) Controlled delivery of water soluble and hydrolytically unstable anti-cancer drugs for polymeric implants. *Polymer Preprints* **32**: 219–220
Elliott TE, Buckner JC and Cascino TL (1991) Phase II study of ifosfamide with mesna in adult patients with recurrent diffuse astrocytoma. *J Neuro-Oncol* **10**: 27–30
Elliott TE, Dinapoli RP, O'Fallon JR, et al (1997) Randomized trial of radiation therapy (RT) plus dibromodulcitol (DBD) versus RT plus BCNU in high-grade astrocytoma. *J Neuro-Oncol* **33**: 239–250
EORTC Brain Tumor Cooperative Group (1985) Effect of AZQ(1,4 Cyclohexadiene-1,4-diacarbamic-acid-2,5-bis 1-aziridynyl-d3, 6 dioxodiethylester) in recurring supratentorial malignant brain gliomas: a phase II study. *Eur J Clin Oncol* **21**: 143–146
Eyre HJ, Quagliana JM, Eltringham JR, et al (1983) Randomized comparisons of radiotherapy and CCNU versus radiotherapy, CCNU plus procarbazine for the treatment of malignant gliomas following surgery. *J Neuro-Oncol* **1**: 171–177
Fine HA and Antman KH (1992) High-dose chemotherapy with autologous bone marrow transplantation in the treatment of high grade astrocytomas in adults: therapeutic rationale and clinical experience. *Bone Marrow Transplant* **10**: 315–321
Fine HA, Dear KBG, Loeffler JS, et al (1993) Meta-analysis of radiation therapy with and without adjuvant chemotherapy for malignant gliomas in adults. *Cancer* **71**: 2585–2597
Fine HA, Figg WD, Jaeckle K, et al (2000) Phase II trials of the antiangiogenic agent thalidomide in patient with recurrent high-grade gliomas. *J Clin Oncol* **18**: 708-715
Folkman J and Klagsbrun M (1987) Angiogenic factors. *Science* **235**: 442–447
Ford J, Osborn C, Barton T and Bleehen NM (1998) A phase I study of intravenous RMP-7 with carboplatin in patients with progression of malignant glioma. *Eur J Cancer* **34**: 1807–1811
Friedman HS, Petros WP, Friedman AH, et al (1999) Irinotecan therapy in adults with recurrent or progressive malignant glioma. *J Clin Oncol* **17**: 1516–1525
Fulling K and Garcia D (1985) Anaplastic astrocytoma of the adult cerebrum; prognostic value of histologic features. *Cancer* **55**: 928–931
Galanis E, Buckner JC, Schomberg PJ, et al (1997) Effective chemotherapy for advanced CNS embryonal tumors in adults. *J Clin Oncol* **15**: 2939–2944
Galanis E, Buckner JC, Burch PA, et al (1998) Phase II trial of nitrogen mustard, vincristine, and procarbazine in patients with recurrent glioma: North Central Cancer Treatment Group Results. *J Clin Oncol* (in press)
Germain RN and Margulies DH (1993) The biochemistry and cell biology of antigen processing and presentation. *Annu Rev Immunol* **11**: 403–450
Giannone L and Wolff S (1987) Phase II treatment of central nervous system gliomas with high-dose etoposide and autologous bone marrow transplantation. *Cancer Treat Rep* **71**: 759–761
Gilbert MR, Lunsford LD, Kondziolka D, et al (1993) A phase II trial of continuous infusion chemotherapy, external beam radiotherapy and local boots radiotherapy for malignant gliomas. *Proc Am Soc Clin Oncol* **12**: 176
Glass J, Hochberg FH, Gruber ML, et al (1992) The treatment of oligodendrogliomas and mixed oligodendroglioma-astrocytomas with PCV chemotherapy. *J Neurosurg* **76**: 741
Grant R, Slattery J, Gregor A, et al (1994) Recording neurological impairment in clinical trials of glioma. *J Neuro-Oncol* **19**: 37–49
Green SB, Byar DP, Walker MD, et al (1983) Comparisons of carmustine procarbazine and high-dose methylprednisolone as additions to surgery and radiotherapy for the treatment of malignant glioma. *Cancer Treat Rep* **67**: 121
Grossman SA, Wharam M, Sheidler V, et al (1997) Phase II study of continuous infusion carmustine and cisplatin followed by cranial irradiation in adults with newly diagnosed high-grade astrocytoma. *J Clin Oncol* **15**: 2596–2603
Grossman SA, Hochberg F, Fisher J, et al (1998) Increased 9-aminocamptothecin dose requirements in patients on anticonvulsants. NABTT CNS Consortium. The new approaches to brain tumor therapy. *Cancer Chemother Pharmacol* **432**: 118–126
Gutin PH, Wilson CB and Kumar AR (1975) Phase II study of procarbazine, CCNU and vincristine combination chemotherapy in the treatment of malignant brain tumors. *Cancer* **35**: 1398–1404
Hoogstraten B, Gottlieb JA and Caoili E (1973) CCNU (1-(2-chloroethyl)-3-cyclohexyl-1-nitrosourea, NSC-79037) in the treatment of cancer: phase II study. *Cancer* **32**: 38–43
Jeremic B, Grujmicic D, Jevremovic S, et al (1992) Carboplatin and etoposide chemotherapy regimen for recurrent malignant glioma: a phase II study. *J Clin Oncol* **10**: 1074–1079
Karp DD, Silberman SL, Csudae R, et al (1999) Phase I dose escalation study of epidermal growth factor receptor (EGFR) tyrosine kinase (TK) inhibitor CP-358,774 in patients with advanced solid tumors. *Proc Am Soc Clin Oncol* **18**: 388a
Kaye A and Laws E (ed) (1995) *Brain Tumors: An Encyclopedic Approach*. Churchill Livingstone: New York
Kessinger A, Lemon HM and Foley JF (1979) VM-26 as a second drug in the treatment of brain gliomas. *Cancer Treat Rep* **63**: 511–512
Kohl NE, Mosser SD, deSolms J, et al (1993) Selective inhibition of ras-dependent transformation by a farnesyltransferase inhibitor. *Science* **260**: 1934
Krishna Narla R, Liu X-P, Klis D and Uckun FM (1998) Inhibition of human glioblastoma cell adhesion and invasion by 4-(4′-hydroxylphenyl)-amino-6,7-dimethoxyquinazoline (WHI-P131) and 4-(3′-Bromo-4′-hydroxylphenyl)-amino-6,7-dimethoxyquinazoline (WHI-P154). *Clin Cancer Res* **4**: 2463–2471
Kyritsis AP, Yung WKA, Bruner J, Gleason MJ and Levin VA (1993) The treatment of anaplastic oligodendrogliomas and mixed gliomas. *Neurosurgery* **32**: 365
Lampert K, Machein U, Machein MR, Conca W, Peter HH and Volk B (1998) Expression of matrix metalloproteinases and their tissue inhibitors in human brain tumors. *Am J Pathol* **153**: 429–437
Lesser GJ and Grossman S (1994) The chemotherapy of high-grade astrocytomas. *Semin Oncol* **21**: 220–235
Levin VA, Edwards MS, Wright DC, et al (1980) Modified procarbazine, CCNU, and vincristine (PCV 3) combination chemotherapy in the treatment of malignant brain tumors. *Cancer Treat Rep* **64**: 237–241
Levin VA, Silver P, Hannigan J, et al (1990) Superiority of post-radiotherapy adjuvant chemotherapy with CCNU, procarbazine, and vincristine (PCV) over BCNU for anaplastic gliomas: NCOG 6G61 final report. *Int J Radiat Oncol Biol Phys* **18**: 321–324
Levin VA (2000) Phase II glioma trails with temozolomide combinations and other new agents. *Cancer Invest* **18**: 95-96
Li V, Folkerth R, Watanabe H, Yu C, Rupnick M, Barnes P, Scott R, Black P, et al (1994) Microvessel count and cerebrospinal fluid basic fibroblast growth factor in children with brain tumors. *Lancet* **344**: 82–86
Luse S (1960) Electron microscopic studies of brain tumors. *Neurology* **10**: 881–905
Macdonald DR, Gaspar LE and Cairncross JG (1990) Successful chemotherapy for newly diagnosed aggressive oligodendroglioma. *Ann Neurol* **27**: 573–574
Mak M, Fung L, Strasser JF, et al (1995) Distribution of drugs following controlled delivery to the brain interstitium. *J Neuro-Oncol* **26**: 91–102
Markert JM, Medlock M, Gillespie GY, et al (1999) Preliminary report on the use of a genetically-engineered HSV-1 in the treatment of malignant glioma in humans. *Neuro-Oncol* **1**: S106
Mineta T, Rabkin SD, Yazaki T, et al (1995) Attenuated multi-mutated herpes simplex virus-1 for the treatment of malignant gliomas. *Nat Med* **1**: 938–943
Mortimer JE, Crowley J, Eyre H, et al (1992) A phase II randomized study comparing sequential and combined intraarterial cisplatin and radiation therapy in primary brain tumors. *A Southwest Oncology Group study*. **69**: 1220–1223
Nelson DF, Diener-West M, Weinstein AS, et al (1986) A randomized comparison of misonidazole sensitized radiotherapy plus BCNU and radiotherapy plus BCNU for treatment of malignant glioma after surgery: final report of an RTOG study. *Int J Radiat Oncol Biol Phys* **12**: 1793
Nelson DF, Diener-West M, Horton J, et al (1988) Combined modality approach to treatment of malignant gliomas – re-evaluation of RTOG 7401/ECOG 1374 with long-term follow-up: a joint study of Radiation Therapy Oncology Group and Eastern Cooperative Oncology Group. *NCI Monogr* **6**: 279–284

© 2000 Cancer Research Campaign 

Newlands ES, Blackledge GRP, Slack JA, et al (1992) Phase I trial of temozolomide (CCRG 81045: M & B 3983: NSC 262856). *Br J Cancer* **65**: 287–291

Newlands ES, O'Reilly SM and Glaser MG (1996) The Charing Cross Hospital experience with temozolomide in patients with gliomas. *Eur J Cancer* **32A**: 2236–2241

Newlands ES, Stevens MFG, Wedge SR, et al (1997*a*) Temozolomide: a review of its discovery, chemical properties, preclinical development and clinical trials. *Cancer Treat* **23**: 35–61

Newlands ES, Wedge SR, Porteous JK, et al (1997*b*) Temozolomide: drug scheduling in vivo and in combination with radiation. *Proc Am Soc Clin Oncol* **16**: 393

O'Reilly MS (1997) Angiostatin. An endogenous inhibitor of angiogenesis and of tumor growth. In: *Regulation of Angiogenesis*, Goldberg ID and Rosen EM (eds). Birkhauser Verlag: Basel, Switzerland

O'Reilly MS, Boehm T, Shing Y, Fukai N, Vasios G, Lane WS, et al (1997) Endostatin: an endogenous inhibitor of angiogenesis and tumor growth. *Cell* **88**: 277–285

Oldfield EH, Ram Z, Culver KW, et al (1993) Gene therapy for the treatment of brain tumors using intra-tumoral transduction with the thymidine kinase gene and intravenous ganciclovir. *Hum Gene Ther* **4**: 39–69

Osaba D, Aaronson N, Sneeuw K, et al (1996) The development and psychometric validation of a brain cancer quality-of-life questionnaire for use in combination with general cancer specific questionnaires. *Qual Life Res* **5**: 139–150

Petersdorf SH and Berger MS (1996) Concepts in neurosurgery: the molecular basis of neurosurgical disease. In: *Molecular Basis of Chemotherapy for Brain Tumors*, Raffel C and Harsh GR (eds) pp. 198–210. Williams and Wilkins: Baltimore

Phillips G, Wolff S, Fay J, et al (1986) Intensive 1,3-bis (2-chloroethyl)-1-nitrosourea (BCNU) monochemotherapy and autologous marrow transplantation for malignant glioma. *J Clin Oncol* **4**: 639–645

Plate K, Breier G, Weich H and Risau W (1994) Vascular endothelial growth factor and glioma angiogenesis: coordinate induction of VEGF receptors, distribution of VEGF protein and possible in vivo regulatory mechanisms. *Int J Cancer* **59**: 520–529

Prados M, Scott C, Sandler H, et al (1997) Phase III randomized study of radiotherapy with or without PCV for the treatment of anaplastic astrocytoma: RTOG 9404 interim report. *Proc ASTRO* 138

Prados M, Yung A, Chang S, et al (1999) A phase 2 trial of Temodal® (temozolomide) in patients with anaplastic astrocytoma at first relapse. *Proc Am Soc Clin Oncol* **18**: 139a

Rodriguez LA, Prados M and Silver P (1989) Revaluation of procarbazine for the treatment of recurrent malignant central nervous system tumours. *Cancer* **64**: 2420–2423

Russell D and Rubinstein L (1989) *Pathology of Tumors of the Nervous System*. Williams & Wilkins: Baltimore

Safgren SL, Reid JM, Buckner JC, Rajkumar SV, Rios R and Ames MM (1998) Pharmacokinetics of dacarbazine and its active metabolites MTIC and HMMTIC in patients with recurrent glioma. *Proc Am Assoc Cancer Res* **39**: 327

Sanson M, Ameri A, Monjour A, et al (1996) Treatment of recurrent malignant supratentorial gliomas with ifosfamide, carboplatin, and etoposide: a phase II study. *Eur J Cancer* **32A**: 2229–2235

Sebag-Montefiore DJ, Douek E, Kingston JE, et al (1992) Intracranial germ cell tumours: I. Experience with platinum-based chemotherapy and implications for curative chemoradiotherapy. *Clin Oncol* **4**: 345–350

Shapiro WR, Green SB, Burger PC, et al (1989) Randomized trial of three chemotherapy regimens and two radiotherapy regimens in postoperative treatment of malignant glioma: Brain Tumor Cooperative Group trial 8001. *J Neurosurg* **71**: 1

Shapiro WR, Green SB, Burger PC, et al (1992) A randomized comparison of intra-arterial versus intravenous BCNU, with or without intravenous 5-fluorouracil, for newly diagnosed patients with malignant gliomas. *J Neurosurg* **76**: 772

Siu LL, Hidalgo M, Nemunaitis J, et al (1999) Dose and schedule-duration escalation of the epidermal growth factor receptor (EGFR) tyrosine kinase (TK) inhibitor CP-358, 774: a phase I and pharmacokinetic (PK) study. *Proc Am Soc Clin Oncol* **18**: 388a

Spence AM, Berger MS, Livingston RB, et al (1992) Phase II evaluation of high-dose intravenous cisplatin for treatment of adult malignant gliomas recurrent after chloroethylnitrosourea failure. *J Neuro-Oncol* **12**: 187–191

Takamiya Y, Brem H, Ojeifo J, Mineta T and Martuza RL (1994) AGM-1470 inhibits the growth of human glioblastoma cells in vitro and in vivo. *Neurosurg* **34**: 869–875

Tirelli U, D'Incalci M and Canetta M (1984) Etoposide (VP-16-213) in malignant brain tumors: a phase II study. *J Clin Oncol* **2**: 432–437

Walker MD and Hurwitz BS (1970) BCNU 1,3-bis (2-chloroethyl)-1-nitrosourea, (NSC-409962) in the treatment of malignant brain tumor. A preliminary report. *Cancer Chemother Rep* **54**: 263–271

Walker MD, Alexander E, Hunt WE, et al (1978) Evaluation of BCNU and/or radiotherapy in the treatment of anaplastic gliomas: a cooperative clinical trial. *J Neurosurg* **49**: 333

Walker MD, Green SB, Byar DP, et al (1980) Randomized comparisons of radiotherapy and nitrosoureas for the treatment of malignant glioma after surgery. *N Engl J Med* **303**: 1323

Weber F, Floeth F, Rommel F (1999) Topic treatment of malignant glioma by intratumoral administration of IL-4-toxin. *Neuro-Oncol* **1**: S105

Wilson CB, Bodrey EB and Enot KJ (1970) 1,3-bis (2-chloroethyl)-1-nitrosourea (NSC-409962) in the treatment of brain tumors. *Cancer Chemother Rep* **54**: 273–281

Witters L, Kumar R, Mandal M, et al (1997) Antisense oligonucleotides to the epidermal growth factor receptor. *Proc Am Assoc Cancer Res* **38**: 315

Yoneda T, Lyall RM, Alsina MM, et al (1991) The antiproliferative effects of tyrosine kinase inhibitors tyrphostins on a human squamous cell carcinoma in vitro in the nude mice. *Cancer Res* **51**: 4430

Yoshida S, Tanaka R, Takai N and Ono K (1990) Adoptive immunotherapy with LAK cells and interleukin-2 in the treatment of recurrent malignant gliomas. *Curr Ther Res* **47**: 654

Yung WK, Harris MI and Bruner JM (1989) Intravenous BCNU and AZQ in patients with recurrent malignant gliomas. *J Neuro-Oncol* **7**: 237–240

Yung WK, Mechtler L and Gleason MJ (1991) Intravenous carboplatin for recurrent malignant glioma: a phase II study. *J Clin Oncol* **9**: 860–864

Yung A, Levén VA, Albright R, et al (1999) Randomized trial of temodal (TEM) vs. procarbazine (PCB) in glioblastoma multiforme (GBM) at first relapse. *Proc Am Soc Clin Oncol* **18**: 139a

 *© 2000 Cancer Research Campaign*

## References

[PDF_00782] Ameri A., Poisson M., Chen Q. M., Delattre J. Y. (1993). Treatment of recurrent malignant supratentorial gliomas with the association of procarbazine, thiotepa and vincristine: a phase II study.. J Neurooncol.

[PDF_00785] Barba D., Saris S. C., Holder C., Rosenberg S. A., Oldfield E. H. (1989). Intratumoral LAK cell and interleukin-2 therapy of human gliomas.. J Neurosurg.

[PDF_00787] Belanich M., Pastor M., Randall T., Guerra D., Kibitel J., Alas L., Li B., Citron M., Wasserman P., White A. (1996). Retrospective study of the correlation between the DNA repair protein alkyltransferase and survival of brain tumor patients treated with carmustine.. Cancer Res.

[PDF_00795] Bischoff J. R., Kirn D. H., Williams A., Heise C., Horn S., Muna M., Ng L., Nye J. A., Sampson-Johannes A., Fattaey A. (1996). An adenovirus mutant that replicates selectively in p53-deficient human tumor cells.. Science.

[PDF_00801] Bower M., Newlands E. S., Bleehen N. M., Brada M., Begent R. J., Calvert H., Colquhoun I., Lewis P., Brampton M. H. (1997). Multicentre CRC phase II trial of temozolomide in recurrent or progressive high-grade glioma.. Cancer Chemother Pharmacol.

[PDF_00804] Brada M., Sharpe G. (1996). Chemotherapy of high-grade gliomas: beginning of a new era or the end of the old?. Eur J Cancer.

[PDF_00806] Brady L. W., Miyamoto C., Woo D. V., Rackover M., Emrich J., Bender H., Dadparvar S., Steplewski Z., Koprowski H., Black P. (1992). Malignant astrocytomas treated with iodine-125 labeled monoclonal antibody 425 against epidermal growth factor receptor: a phase II trial.. Int J Radiat Oncol Biol Phys.

[PDF_00812] Brem H., Piantadosi S., Burger P. C., Walker M., Selker R., Vick N. A., Black K., Sisti M., Brem S., Mohr G. (1995). Placebo-controlled trial of safety and efficacy of intraoperative controlled delivery by biodegradable polymers of chemotherapy for recurrent gliomas. The Polymer-brain Tumor Treatment Group.. Lancet.

[PDF_00823] Buckner J. C., Brown L. D., Cascino T. L., Gerstner J. B., Krook J. E., Westberg M. W., Wiesenfeld M., O'Fallon J. R., Scheithauer B. (1990). Phase II evaluation of infusional etoposide and cisplatin in patients with recurrent astrocytoma.. J Neurooncol.

[PDF_00826] Burger P. C., Vogel F. S., Green S. B., Strike T. A. (1985). Glioblastoma multiforme and anaplastic astrocytoma. Pathologic criteria and prognostic implications.. Cancer.

[PDF_00837] Cairncross G., Macdonald D., Ludwin S., Lee D., Cascino T., Buckner J., Fulton D., Dropcho E., Stewart D., Schold C. (1994). Chemotherapy for anaplastic oligodendroglioma. National Cancer Institute of Canada Clinical Trials Group.. J Clin Oncol.

[PDF_00833] Cairncross J. G., Macdonald D. R. (1991). Chemotherapy for oligodendroglioma. Progress report.. Arch Neurol.

[PDF_00835] Cairncross J. G., Macdonald D. R., Ramsay D. A. (1992). Aggressive oligodendroglioma: a chemosensitive tumor.. Neurosurgery.

[PDF_00839] Cascino T. L., Brown L. D., Morton R. F., Everson L. K., Marschke R. F., Dinapoli R. P., O'Fallon J. R. (1988). Evaluation of fludarabine phosphate in patients with recurrent glioma.. Am J Clin Oncol.

[PDF_00841] Chamberlain M. C., Kormanik P. (1995). Salvage chemotherapy with paclitaxel for recurrent primary brain tumors.. J Clin Oncol.

[PDF_00843] Chamberlain M. C., Prados M. D., Silver P., Levin V. A. (1988). A phase II trial of oral melphalan in recurrent primary brain tumors.. Am J Clin Oncol.

[PDF_00845] Chang C. H., Horton J., Schoenfeld D., Salazer O., Perez-Tamayo R., Kramer S., Weinstein A., Nelson J. S., Tsukada Y. (1983). Comparison of postoperative radiotherapy and combined postoperative radiotherapy and chemotherapy in the multidisciplinary management of malignant gliomas. A joint Radiation Therapy Oncology Group and Eastern Cooperative Oncology Group study.. Cancer.

[PDF_00848] Chang S. M., Kuhn J. G., Rizzo J., Robins H. I., Schold S. C., Spence A. M., Berger M. S., Mehta M. P., Bozik M. E., Pollack I. (1998). Phase I study of paclitaxel in patients with recurrent malignant glioma: a North American Brain Tumor Consortium report.. J Clin Oncol.

[PDF_00854] Cohadon F., Aouad N., Rougier A., Vital C., Rivel J., Dartigues J. F. (1985). Histologic and non-histologic factors correlated with survival time in supratentorial astrocytic tumors.. J Neurooncol.

[PDF_00863] Coyle T., Baptista J., Winfield J., Clark K., Poiesz B., Kirshner J., Scalzo A., Newman-Palmer N., King R., Graziano S. (1990). Mechlorethamine, vincristine, and procarbazine chemotherapy for recurrent high-grade glioma in adults: a phase II study.. J Clin Oncol.

[PDF_00866] D'Amato R. J., Loughnan M. S., Flynn E., Folkman J. (1994). Thalidomide is an inhibitor of angiogenesis.. Proc Natl Acad Sci U S A.

[PDF_00868] Daumas-Duport C., Scheithauer B., O'Fallon J., Kelly P. (1988). Grading of astrocytomas. A simple and reproducible method.. Cancer.

[PDF_00870] Deutsch M., Green S. B., Strike T. A., Burger P. C., Robertson J. T., Selker R. G., Shapiro W. R., Mealey J., Ransohoff J., Paoletti P. (1989). Results of a randomized trial comparing BCNU plus radiotherapy, streptozotocin plus radiotherapy, BCNU plus hyperfractionated radiotherapy, and BCNU following misonidazole plus radiotherapy in the postoperative treatment of malignant glioma.. Int J Radiat Oncol Biol Phys.

[PDF_00875] Dietrich P. Y., Walker P. R., Saas P., de Tribolet N. (1997). Immunobiology of gliomas: new perspectives for therapy.. Ann N Y Acad Sci.

[PDF_00877] Dinapoli R. P., Brown L. D., Arusell R. M., Earle J. D., O'Fallon J. R., Buckner J. C., Scheithauer B. W., Krook J. E., Tschetter L. K., Maier J. A. (1993). Phase III comparative evaluation of PCNU and carmustine combined with radiation therapy for high-grade glioma.. J Clin Oncol.

[PDF_00880] Dolan M. E., Pegg A. E. (1997). O6-benzylguanine and its role in chemotherapy.. Clin Cancer Res.

[PDF_00886] Elliott T. E., Buckner J. C., Cascino T. L., Levitt R., O'Fallon J. R., Scheithauer B. W. (1991). Phase II study of ifosfamide with mesna in adult patients with recurrent diffuse astrocytoma.. J Neurooncol.

[PDF_00889] Elliott T. E., Dinapoli R. P., O'Fallon J. R., Krook J. E., Earle J. D., Morton R. F., Levitt R., Tschetter L. K., Scheithauer B. W., Pfeifle D. M. (1997). Randomized trial of radiation therapy (RT) plus dibromodulcitol (DBD) versus RT plus BCNU in high grade astrocytoma.. J Neurooncol.

[PDF_00896] Eyre H. J., Quagliana J. M., Eltringham J. R., Frank J., O'Bryan R. M., McDonald B., Rivkin S. E. (1983). Randomized comparisons of radiotherapy and CCNU versus radiotherapy, CCNU plus procarbazine for the treatment of malignant gliomas following surgery. A Southwest Oncology Group Report.. J Neurooncol.

[PDF_00900] Fine H. A., Antman K. H. (1992). High-dose chemotherapy with autologous bone marrow transplantation in the treatment of high grade astrocytomas in adults: therapeutic rationale and clinical experience.. Bone Marrow Transplant.

[PDF_00904] Fine H. A., Dear K. B., Loeffler J. S., Black P. M., Canellos G. P. (1993). Meta-analysis of radiation therapy with and without adjuvant chemotherapy for malignant gliomas in adults.. Cancer.

[PDF_00907] Fine H. A., Figg W. D., Jaeckle K., Wen P. Y., Kyritsis A. P., Loeffler J. S., Levin V. A., Black P. M., Kaplan R., Pluda J. M. (2000). Phase II trial of the antiangiogenic agent thalidomide in patients with recurrent high-grade gliomas.. J Clin Oncol.

[PDF_00910] Folkman J., Klagsbrun M. (1987). Angiogenic factors.. Science.

[PDF_00911] Ford J., Osborn C., Barton T., Bleehen N. M. (1998). A phase I study of intravenous RMP-7 with carboplatin in patients with progression of malignant glioma.. Eur J Cancer.

[PDF_00914] Friedman H. S., Petros W. P., Friedman A. H., Schaaf L. J., Kerby T., Lawyer J., Parry M., Houghton P. J., Lovell S., Rasheed K. (1999). Irinotecan therapy in adults with recurrent or progressive malignant glioma.. J Clin Oncol.

[PDF_00916] Fulling K. H., Garcia D. M. (1985). Anaplastic astrocytoma of the adult cerebrum. Prognostic value of histologic features.. Cancer.

[PDF_00918] Galanis E., Buckner J. C., Schomberg P. J., Hammack J. E., Raffel C., Scheithauer B. W. (1997). Effective chemotherapy for advanced CNS embryonal tumors in adults.. J Clin Oncol.

[PDF_00925] Giannone L., Wolff S. N. (1987). Phase II treatment of central nervous system gliomas with high-dose etoposide and autologous bone marrow transplantation.. Cancer Treat Rep.

[PDF_00931] Glass J., Hochberg F. H., Gruber M. L., Louis D. N., Smith D., Rattner B. (1992). The treatment of oligodendrogliomas and mixed oligodendroglioma-astrocytomas with PCV chemotherapy.. J Neurosurg.

[PDF_00934] Grant R., Slattery J., Gregor A., Whittle I. R. (1994). Recording neurological impairment in clinical trials of glioma.. J Neurooncol.

[PDF_00936] Green S. B., Byar D. P., Walker M. D., Pistenmaa D. A., Alexander E., Batzdorf U., Brooks W. H., Hunt W. E., Mealey J., Odom G. L. (1983). Comparisons of carmustine, procarbazine, and high-dose methylprednisolone as additions to surgery and radiotherapy for the treatment of malignant glioma.. Cancer Treat Rep.

[PDF_00942] Grossman S. A., Hochberg F., Fisher J., Chen T. L., Kim L., Gregory R., Grochow L. B., Piantadosi S. (1998). Increased 9-aminocamptothecin dose requirements in patients on anticonvulsants. NABTT CNS Consortium. The New Approaches to Brain Tumor Therapy.. Cancer Chemother Pharmacol.

[PDF_00939] Grossman S. A., Wharam M., Sheidler V., Kleinberg L., Zeltzman M., Yue N., Piantadosi S. (1997). Phase II study of continuous infusion carmustine and cisplatin followed by cranial irradiation in adults with newly diagnosed high-grade astrocytoma.. J Clin Oncol.

[PDF_00946] Gutin P. H., Wilson C. B., Kumar A. R., Boldrey E. B., Levin V., Powell M., Enot K. J. (1975). Phase II study of procarbazine, CCNU, and vincristine combination chemotherapy in the treatment of malignant brain tumors.. Cancer.

[PDF_00949] Hoogstraten B., Gottlieb J. A., Caoili E., Tucker W. G., Talley R. W., Haut A. (1973). CCNU (1-(2-chloroethyl)-3-cyclohexyl-1-nitrosourea, NSC-79037) in the treatment of cancer. Phase II study.. Cancer.

[PDF_00952] Jeremic B., Grujicic D., Jevremovic S., Stanisavljevic B., Milojevic L., Djuric L., Mijatovic L. (1992). Carboplatin and etoposide chemotherapy regimen for recurrent malignant glioma: a phase II study.. J Clin Oncol.

[PDF_00961] Kessinger A., Lemon H. M., Foley J. F. (1979). VM-26 as a second drug in the treatment of brain gliomas.. Cancer Treat Rep.

[PDF_00963] Kohl N. E., Mosser S. D., deSolms S. J., Giuliani E. A., Pompliano D. L., Graham S. L., Smith R. L., Scolnick E. M., Oliff A., Gibbs J. B. (1993). Selective inhibition of ras-dependent transformation by a farnesyltransferase inhibitor.. Science.

[PDF_00969] Kyritsis A. P., Yung W. K., Bruner J., Gleason M. J., Levin V. A. (1993). The treatment of anaplastic oligodendrogliomas and mixed gliomas.. Neurosurgery.

[PDF_00989] LUSE S. A. (1960). Electron microscopic studies of brain tumors.. Neurology.

[PDF_00972] Lampert K., Machein U., Machein M. R., Conca W., Peter H. H., Volk B. (1998). Expression of matrix metalloproteinases and their tissue inhibitors in human brain tumors.. Am J Pathol.

[PDF_00975] Lesser G. J., Grossman S. (1994). The chemotherapy of high-grade astrocytomas.. Semin Oncol.

[PDF_00977] Levin V. A., Edwards M. S., Wright D. C., Seager M. L., Schimberg T. P., Townsend J. J., Wilson C. B. (1980). Modified procarbazine, CCNU, and vincristine (PCV 3) combination chemotherapy in the treatment of malignant brain tumors.. Cancer Treat Rep.

[PDF_00980] Levin V. A., Silver P., Hannigan J., Wara W. M., Gutin P. H., Davis R. L., Wilson C. B. (1990). Superiority of post-radiotherapy adjuvant chemotherapy with CCNU, procarbazine, and vincristine (PCV) over BCNU for anaplastic gliomas: NCOG 6G61 final report.. Int J Radiat Oncol Biol Phys.

[PDF_00986] Li V. W., Folkerth R. D., Watanabe H., Yu C., Rupnick M., Barnes P., Scott R. M., Black P. M., Sallan S. E., Folkman J. (1994). Microvessel count and cerebrospinal fluid basic fibroblast growth factor in children with brain tumours.. Lancet.

[PDF_00990] Macdonald D. R., Gaspar L. E., Cairncross J. G. (1990). Successful chemotherapy for newly diagnosed aggressive oligodendroglioma.. Ann Neurol.

[PDF_00992] Mak M., Fung L., Strasser J. F., Saltzman W. M. (1995). Distribution of drugs following controlled delivery to the brain interstitium.. J Neurooncol.

[PDF_00997] Mineta T., Rabkin S. D., Yazaki T., Hunter W. D., Martuza R. L. (1995). Attenuated multi-mutated herpes simplex virus-1 for the treatment of malignant gliomas.. Nat Med.

[PDF_00999] Mortimer J. E., Crowley J., Eyre H., Weiden P., Eltringham J., Stuckey W. J. (1992). A phase II randomized study comparing sequential and combined intraarterial cisplatin and radiation therapy in primary brain tumors. A Southwest Oncology Group study.. Cancer.

[PDF_00965] Narla R. K., Liu X. P., Klis D., Uckun F. M. (1998). Inhibition of human glioblastoma cell adhesion and invasion by 4-(4'-hydroxylphenyl)-amino-6,7-dimethoxyquinazoline (WHI-P131) and 4-(3'-bromo-4'-hydroxylphenyl)-amino-6,7-dimethoxyquinazoline (WHI-P154).. Clin Cancer Res.

[PDF_01006] Nelson D. F., Diener-West M., Horton J., Chang C. H., Schoenfeld D., Nelson J. S. (1988). Combined modality approach to treatment of malignant gliomas--re-evaluation of RTOG 7401/ECOG 1374 with long-term follow-up: a joint study of the Radiation Therapy Oncology Group and the Eastern Cooperative Oncology Group.. NCI Monogr.

[PDF_01002] Nelson D. F., Diener-West M., Weinstein A. S., Schoenfeld D., Nelson J. S., Sause W. T., Chang C. H., Goodman R., Carabell S. (1986). A randomized comparison of misonidazole sensitized radiotherapy plus BCNU and radiotherapy plus BCNU for treatment of malignant glioma after surgery: final report of an RTOG study.. Int J Radiat Oncol Biol Phys.

[PDF_01012] Newlands E. S., Blackledge G. R., Slack J. A., Rustin G. J., Smith D. B., Stuart N. S., Quarterman C. P., Hoffman R., Stevens M. F., Brampton M. H. (1992). Phase I trial of temozolomide (CCRG 81045: M&B 39831: NSC 362856).. Br J Cancer.

[PDF_01014] Newlands E. S., O'Reilly S. M., Glaser M. G., Bower M., Evans H., Brock C., Brampton M. H., Colquhoun I., Lewis P., Rice-Edwards J. M. (1996). The Charing Cross Hospital experience with temozolomide in patients with gliomas.. Eur J Cancer.

[PDF_01017] Newlands E. S., Stevens M. F., Wedge S. R., Wheelhouse R. T., Brock C. (1997). Temozolomide: a review of its discovery, chemical properties, pre-clinical development and clinical trials.. Cancer Treat Rev.

[PDF_01025] O'Reilly M. S., Boehm T., Shing Y., Fukai N., Vasios G., Lane W. S., Flynn E., Birkhead J. R., Olsen B. R., Folkman J. (1997). Endostatin: an endogenous inhibitor of angiogenesis and tumor growth.. Cell.

[PDF_01028] Oldfield E. H., Ram Z., Culver K. W., Blaese R. M., DeVroom H. L., Anderson W. F. (1993). Gene therapy for the treatment of brain tumors using intra-tumoral transduction with the thymidine kinase gene and intravenous ganciclovir.. Hum Gene Ther.

[PDF_01031] Osoba D., Aaronson N. K., Muller M., Sneeuw K., Hsu M. A., Yung W. K., Brada M., Newlands E. (1996). The development and psychometric validation of a brain cancer quality-of-life questionnaire for use in combination with general cancer-specific questionnaires.. Qual Life Res.

[PDF_01038] Phillips G. L., Wolff S. N., Fay J. W., Herzig R. H., Lazarus H. M., Schold C., Herzig G. P. (1986). Intensive 1,3-bis (2-chloroethyl)-1-nitrosourea (BCNU) monochemotherapy and autologous marrow transplantation for malignant glioma.. J Clin Oncol.

[PDF_01042] Plate K. H., Breier G., Weich H. A., Mennel H. D., Risau W. (1994). Vascular endothelial growth factor and glioma angiogenesis: coordinate induction of VEGF receptors, distribution of VEGF protein and possible in vivo regulatory mechanisms.. Int J Cancer.

[PDF_01052] Rodriguez L. A., Prados M., Silver P., Levin V. A. (1989). Reevaluation of procarbazine for the treatment of recurrent malignant central nervous system tumors.. Cancer.

[PDF_01060] Sanson M., Ameri A., Monjour A., Sahmoud T., Ronchin P., Poisson M., Delattre J. Y. (1996). Treatment of recurrent malignant supratentorial gliomas with ifosfamide, carboplatin and etoposide: a phase II study.. Eur J Cancer.

[PDF_01063] Sebag-Montefiore D. J., Douek E., Kingston J. E., Plowman P. N. (1992). Intracranial germ cell tumours: I. Experience with platinum based chemotherapy and implications for curative chemoradiotherapy.. Clin Oncol (R Coll Radiol).

[PDF_01066] Shapiro W. R., Green S. B., Burger P. C., Mahaley M. S., Selker R. G., VanGilder J. C., Robertson J. T., Ransohoff J., Mealey J., Strike T. A. (1989). Randomized trial of three chemotherapy regimens and two radiotherapy regimens and two radiotherapy regimens in postoperative treatment of malignant glioma. Brain Tumor Cooperative Group Trial 8001.. J Neurosurg.

[PDF_01070] Shapiro W. R., Green S. B., Burger P. C., Selker R. G., VanGilder J. C., Robertson J. T., Mealey J., Ransohff J., Mahaley M. S. (1992). A randomized comparison of intra-arterial versus intravenous BCNU, with or without intravenous 5-fluorouracil, for newly diagnosed patients with malignant glioma.. J Neurosurg.

[PDF_01077] Spence A. M., Berger M. S., Livingston R. B., Ali-Osman F., Griffin B. (1992). Phase II evaluation of high-dose intravenous cisplatin for treatment of adult malignant gliomas recurrent after chloroethylnitrosourea failure.. J Neurooncol.

[PDF_01080] Takamiya Y., Brem H., Ojeifo J., Mineta T., Martuza R. L. (1994). AGM-1470 inhibits the growth of human glioblastoma cells in vitro and in vivo.. Neurosurgery.

[PDF_01083] Tirelli U., D'Incalci M., Canetta R., Tumolo S., Franchin G., Veronesi A., Galligioni E., Trovò M. G., Rossi C., Grigoletto E. (1984). Etoposide (VP-16-213) in malignant brain tumors: a phase II study.. J Clin Oncol.

[PDF_01088] Walker M. D., Alexander E., Hunt W. E., MacCarty C. S., Mahaley M. S., Mealey J., Norrell H. A., Owens G., Ransohoff J., Wilson C. B. (1978). Evaluation of BCNU and/or radiotherapy in the treatment of anaplastic gliomas. A cooperative clinical trial.. J Neurosurg.

[PDF_01091] Walker M. D., Green S. B., Byar D. P., Alexander E., Batzdorf U., Brooks W. H., Hunt W. E., MacCarty C. S., Mahaley M. S., Mealey J. (1980). Randomized comparisons of radiotherapy and nitrosoureas for the treatment of malignant glioma after surgery.. N Engl J Med.

[PDF_01085] Walker M. D., Hurwitz B. S. (1970). BCNU (1,3-bis(2-chloroethyl)-1-nitrosourea; NSC-409962) in the treatment of malignant brain tumor--a preliminary report.. Cancer Chemother Rep.

[PDF_01096] Wilson C. B., Boldrey E. B., Enot K. J. (1970). 1,3-bis (2-chloroethyl)-1-nitrosourea (NSC-409962) in the treatment of brain tumors.. Cancer Chemother Rep.

[PDF_01101] Yoneda T., Lyall R. M., Alsina M. M., Persons P. E., Spada A. P., Levitzki A., Zilberstein A., Mundy G. R. (1991). The antiproliferative effects of tyrosine kinase inhibitors tyrphostins on a human squamous cell carcinoma in vitro and in nude mice.. Cancer Res.

[PDF_01107] Yung W. K., Harris M. I., Bruner J. M., Feun L. G. (1989). Intravenous BCNU and AZQ in patients with recurrent malignant gliomas.. J Neurooncol.

[PDF_01109] Yung W. K., Mechtler L., Gleason M. J. (1991). Intravenous carboplatin for recurrent malignant glioma: a phase II study.. J Clin Oncol.

